# Content-based validity evidence of the Acute Voice Fatigue Self-Perception Scale (AFA-Voz Scale)

**DOI:** 10.1590/2317-1782/e20250060en

**Published:** 2026-04-10

**Authors:** Priscila Oliveira, Mara Behlau

**Affiliations:** 1 Departamento de Fonoaudiologia, Universidade Federal da Paraíba – UFPB - João Pessoa (PB), Brasil.; 2 Universidade Federal de São Paulo – UNIFESP - São Paulo (SP), Brasil.; 3 Centro de Estudos da Voz – CEV - São Paulo (SP), Brasil.

**Keywords:** Voice, Fatigue, Self-Assessment, Signs and Symptoms, Validation Study

## Abstract

**Purpose:**

Obtaining evidence of content-based validity of the Self-Perceived Acute Voice Fatigue Scale (AFA-Voz Scale).

**Methods:**

This study followed the Standards for Educational and Psychological Testing (SEPT) guidelines for test validation and was conducted in two stages. First, the content was developed based on the analysis of two systematic reviews with high quality evidence to identify the main aspects to be assessed in acute vocal fatigue. Then, a committee of five voice experts – all with more than ten years of clinical and scientific experience – was formed to assess the relevance of the selected items, using a Likert-type scale in an online form. Overall content validity indexes (CVI) and item validity indexes (CVI-I) were calculated to assess the relevance of the items.

**Results:**

The data indicated that the clinical presentation of acute vocal fatigue can be assessed in three dimensions: the level of self-reported fatigue, physical discomfort, and restriction in voice use. These items were included in the scale, which also included an anamnesis section to characterize the patient and their vocal demands. In the first round of evaluation, a CVI of 0.96 was obtained, with CVI-I ranging from 0.8 to 1.0. After suggested adjustments, the second round presented CVI and CVI-I equal to 1.00.

**Conclusion:**

The items of the AFA-Voz Scale were considered excellent regarding their relevance for the evaluation of acute vocal fatigue. The scale has high potential for clinical use and in future research on the subject.

## INTRODUCTION

Vocal production is an activity characterized by a high degree of muscular interdependence and synergy and is typically not exhausting. However, when subjected to vocal overload, maintaining vocal activity over extended periods or with adequate quality may become difficult, resulting in fatigue. Vocal fatigue is a measurable and highly individual symptom, more frequently reported by professional voice users, with variable impact on the quality of performance across different vocal tasks^(1-3)^. In addition to significantly affecting communication, vocal fatigue also compromises quality of life and professional performance in these individuals^(4)^.

As a complex construct, vocal fatigue lacks a consensual definition in the literature. Nevertheless, most scholars agree in defining it as a debilitating phenomenon that involves vocal demands and individual responses to these demands, encompassing subjective symptoms and a range of clinical manifestations. Accordingly, vocal fatigue may be characterized as a perceived increase in vocal effort accompanied by a decrease in voice quality following prolonged voice use^(5,6)^, or as a quantifiable decline in the physiological performance of voice production or in its perceptual assessment^(1)^.

The term “vocal fatigue” has been broadly used to encompass a variety of symptomatic manifestations, whether acute, episodic, or chronic in nature. “Acute vocal fatigue” refers specifically to a physiological and perceptual state that emerges after a single, intense vocal demand, characterized by a transient threshold of decline in vocal performance, which is usually reversible with vocal rest^(1)^. The absence of monitoring and intervention in acute fatigue conditions contributes to the maintenance or worsening of the initial condition, favoring chronicity and leading to what is characterized as “chronic vocal fatigue.” This progression results in more complex clinical implications and greater communicative, economic, and emotional impairments^(4,7)^.

The scientific literature provides widely recognized instruments for the self-assessment of symptoms related to vocal fatigue, such as the Perceived Phonatory Effort (PPE) scale, the Borg CR-10 Scale, the Voice Handicap Index (VHI), the Vocal Tract Discomfort Scale (VTDS), and the Vocal Fatigue Index (VFI)^(8)^. However, these instruments are limited in their ability to provide a specific measurement of acute vocal fatigue, that is, the immediate response to a vocal demand, highlighting the need for more precise methods for its assessment.

The PPE and the Borg CR-10 scales are subjective effort scales used to quantify an individual’s perception of vocal demand^(9)^. Nevertheless, neither scale accurately differentiates vocal effort from vocal fatigue, as effort may be present even in the absence of fatigue and is influenced by factors such as vocal intensity and environmental conditions. The VHI is a validated questionnaire designed to assess perceived handicap resulting from a voice disorder, but it was not developed specifically to measure vocal fatigue and does not provide a detailed discrimination of the sensation of vocal exhaustion arising from prolonged or intense voice use. The VTDS assesses sensory discomforts in the laryngeal region, such as dryness, burning, and tension. However, these symptoms do not necessarily reflect the presence of vocal fatigue, as they may be associated with other conditions, such as laryngopharyngeal reflux or extralaryngeal muscle tension^(10)^.

The VFI, in turn, was specifically designed for the self-assessment of vocal fatigue but may not be sensitive to its acute manifestation. It is capable of capturing symptoms accumulated over time, with a focus on chronic perceptual aspects frequently associated with repeated voice use over days or weeks, rather than immediate changes following a demanding vocal task^(3)^. Acute vocal fatigue represents a transient response that develops rapidly after a specific vocal overload and may manifest through subtle changes in voice quality, such as increased roughness and greater phonatory effort. These findings may not be accurately captured by the VFI.

Professional voice users may experience acute vocal fatigue during their daily activities or when performing specific vocal tasks, leading to short-term limitations and functional impairment, particularly in professions that require intensive voice use, such as teachers, call center operators, singers, and public speakers. Measuring and monitoring the perception of acute fatigue immediately after performance may provide valuable information regarding the ability to sustain vocal performance, supporting appropriate therapeutic planning for fatigue management, maintenance of optimal professional voice performance, and prevention of chronic voice disorders and/or laryngeal pathologies.

Currently, there are no sensitive and specific instruments in the literature for measuring acute vocal fatigue as perceived by the speaker. Therefore, the development of an instrument specifically aimed at assessing acute vocal fatigue represents an advance in the early screening of functional changes, the monitoring of vocal recovery, and the implementation of more precise and immediate therapeutic interventions.

The objective of this study was to develop and obtain content-based validity evidence for the Acute Voice Fatigue Self-Perception Scale (AFA-Voz), designed for the self-assessment of acute vocal fatigue.

## METHOD

This study was aimed at obtaining content-based validity evidence for a test. To this end, it followed the test validation guidelines described by Pernambuco et al.^(11)^, which present a set of recommendations based on the principles of the Standards for Educational and Psychological Testing. These guidelines were derived from a framework proposed by three North American organizations that compiled the most robust recommendations and definitions related to psychometric aspects in the instrument validation process. The study was approved by the Research Ethics Committee involving human subjects (Approval No. 5,960,551).

Two stages were conducted: (1) content development and (2) evaluation of the relevance of the items that composed the instrument. In Stage 1, two scoping reviews^(8,12)^, with a high level of evidence quality, were consulted to support item selection based on the definition of the construct. Items were selected that, according to the cited studies, could contribute to characterizing and measuring the intensity of acute vocal fatigue.

In Stage 2, a committee of five voice specialists was selected, each with more than 10 years of clinical and/or scientific experience. The experts were informed about the objectives of the study, provided written informed consent, and analyzed the initial version of the proposed scale using an online form. The judges were asked to evaluate the relevance/representativeness of each item using a Likert-type scale, according to the following criteria: 1 = the item is not relevant or not representative; 2 = the item requires major revision to be relevant or representative; 3 = the item requires minor revision to be relevant or representative; and 4 = the item is relevant or representative. When responses 1, 2, or 3 were selected, the judge was requested to provide a suggestion for revising the item to improve its relevance or representativeness.

The overall Content Validity Index (CVI-T) and the Item-Level Content Validity Index (CVI-I) were calculated to determine the level of agreement among judges regarding the relevance or representativeness of the instrument items. The CVI is a method widely used in studies focused on assessing the content validity of a construct, as it measures the proportion of judges who agree on the aspect analyzed in the instrument. This approach allows both individual item analysis and evaluation of the instrument as a whole^(13,14)^.

To calculate the CVI-I, the scores assigned by the evaluators were considered according to the following formula:


$$\frac{CVI-I\;=number\;of\;responses\;rate\;“3”\;or\;“4”}{total\;number\;of\;responses}$$ (1)

The CVI-T was obtained by calculating the simple mean of the CVI-I values related to item relevance, that is, the sum of all individually calculated CVI-I values divided by the number of items included in the evaluation. In this study, the following classification was adopted for interpreting CVI values: excellent (CVI ≥ 0.90), good (0.79 ≥ CVI ≤ 0.89), and poor (CVI ≤ 0.78)^(14)^. Items with values below 0.78 were required to be reformulated or excluded from the protocol.

## RESULTS

During the content development stage, the conceptual definition of acute vocal fatigue was established based on the consulted literature. Acute vocal fatigue was defined as a transient state resulting from recent vocal overload, characterized by increased phonatory effort and a decline in voice quality, with expected recovery after rest. It is distinguished from chronic vocal fatigue by its sudden onset and short duration^(1,4-6)^.

The operational definition considered observable manifestations of vocal fatigue reported in the literature^(8,12)^, as well as the authors’ experience. Three main indicators of acute vocal fatigue were selected: (1) immediately self-reported fatigue level (b); (2) physical discomfort in the laryngeal region, such as tightness, pain, among others (c); and (3) restriction in voice use (d), expressed by frequent pauses or reduced vocal capacity. These three items were included in the initial configuration of the instrument, using a numeric scale (0-10 points) to judge symptom intensity^(8)^. A brief anamnesis section was also added to characterize the participant and their vocal demand (a).

In Stage 2, which involved evaluation of the relevance of the items composing the instrument, two rounds of expert judgment were conducted. In the first round, the overall CVI was 0.96, and the CVI-I values were a = 0.80, b = 1.00, c = 1.00, and d = 1.00, indicating excellent relevance. Across both rounds, no item was considered irrelevant; however, some adjustments were suggested to improve item clarity and objectivity. For item (a), related to the anamnesis section, which included date of data collection, participant name, profession, duration of professional voice use, and type of vocal demand (spoken, sung, or both), the inclusion of items addressing the intensity of the most recent vocal activity (loud, habitual, or soft) and the duration of the most recent vocal activity (in minutes) was suggested. For items (c) and (d), minor rewording of the item descriptions and the inclusion of possible examples were suggested to facilitate symptom perception by the participant, thereby improving interpretation and applicability of the instrument.

In addition to all expert suggestions, an instruction formulated by the authors themselves was incorporated to guide respondents in completing the questionnaire. Accordingly, the final instrument consisted of a brief anamnesis section containing five questions regarding the respondent’s profession and characteristics of vocal demand, in addition to three items specifically addressing the assessment of acute vocal fatigue.

After completion of the second round and implementation of all suggested adjustments, the instrument was re-evaluated, and all CVI and CVI-I values were equal to 1.00 (100.0%), indicating excellent agreement. The final version of the instrument is presented in Chart 1.

**Chart 1:** Final version of the Self-Perception of Acute Voice Fatigue Scale (AFA-Voz Scale)

**ESCALA DE AUTOPERCEPÇÃO DA FADIGA AGUDA PARA A VOZ (Escala AFA-Voz)**									
**Oliveira e Behlau, 2025**									
Nome: _______________________________________________________ Data: ___/___/_____									
Profissão: ____________________________________________________ D.N.: ___/___/_____									
Há quanto tempo usa a voz profissionalmente: _________________________________________									
Tipo de demanda vocal: ( ) Falada ( ) Cantada ( ) Falada e Cantada									
Intensidade da última atividade vocal realizada: ( ) Forte ( ) Habitual ( ) Fraca									
Duração da última atividade vocal realizada (em minutos): ________________________________									
**Instrução: Avalie como você percebe sua voz nesse momento e marque o número que mais representa sua condição atual em relação aos sintomas abaixo:**									
1) **Fadiga**									
De 0 a 10, o quanto você sente sua voz cansada/fadigada agora?									
**1**	**2**	**3**	**4**	**5**	**6**	**7**	**8**	**9**	**10**
Nenhuma fadiga					Máxima fadiga				
*2)* **Desconforto físico**									
De 0 a 10, o quanto você sente de desconforto no pescoço/garganta para produzir a voz agora? *(Por exemplo: aperto, dor, ardência, entre outros.)*									
**1**	**2**	**3**	**4**	**5**	**6**	**7**	**8**	**9**	**10**
Nenhum desconforto					Máximo desconforto				
*3)* **Limitação para usar a voz**									
De 0 a 10, o quanto você sente que sua voz está limitada agora? *(Por exemplo: dificuldade para variar o tom da voz, dificuldade para falar forte, dificuldade para falar fraco, dificuldade para projetar a voz, dificuldade para manter o som, a qualidade ou o timbre da voz, dificuldade para manter o som da voz, dificuldade para continuar falando, dificuldade para continuar cantando, entre outros.)*									
**1**	**2**	**3**	**4**	**5**	**6**	**7**	**8**	**9**	**10**
Nenhuma limitação					Máxima limitação				

## DISCUSSION

In the health sciences, an adequate assessment process depends on the analysis of an individual’s perception of a specific aspect, which can be accomplished through the use of tests whose result interpretations are valid, reliable/precise, and equitable. Content validity represents a stage concerned with gathering evidence related to a test’s topic, wording, format, tasks, or items, as well as the instructions for the procedures required to administer and score it^(11)^. It is an essential step in the development of assessment instruments, as it ensures relevance and comprehensiveness in measuring constructs, resulting in a quantifiable measure of validity^(11,15)^.

In this study, the conceptual and operational definitions of acute vocal fatigue represented an essential stage in the development of the instrument and were grounded in the literature^(8,12)^ and in the authors’ clinical experience. Delimiting the construct and differentiating it from chronic vocal fatigue made it possible to establish more sensitive parameters for detecting acute signs, which have a rapid onset and are reversible with vocal rest. The extensive investigation conducted in the reference studies allows us to state that the findings reflect a synthesis of the most prominent and reliable research evidence on the topic.

The choice of a numerical rating scale (0 to 10 points) was based on its practicality, sensitivity, and widespread acceptance for measuring subjective symptoms such as vocal fatigue. Compared with Likert-type scales, which are designed to measure attitudes or perceptions, numerical rating scales allow the capture of gradual variations in symptom intensity. Although the Visual Analog Scale (VAS) is also sensitive for this purpose and is cited in a large proportion of studies, its application may be limited by the need for visual support and more abstract interpretation, which can compromise response precision. Numerical scales, in contrast, are easy to understand and apply, even in non-clinical settings, while maintaining good assessment accuracy^(16)^.

The experts’ suggestions provided during the judgment rounds were incorporated to improve the clarity and applicability of the instrument. The inclusion of additional items in the anamnesis section, for example, allowed better characterization of the context of recent voice use, while wording adjustments and the inclusion of examples, particularly in item 3, enhanced item comprehension by the judges. In addition, the inclusion of a preliminary instruction contributed to standardizing completion procedures and reducing potential ambiguities in item interpretation.

Although the instrument obtained a satisfactory evaluation in its initial version, the implementation of the suggested adjustments resulted in high agreement among judges regarding item relevance (CVI = 1.00) in the second round. This finding indicates that the selected indicators (immediate self-reported fatigue, physical discomfort in the laryngeal region, and restriction in voice use) were considered representative of the construct under evaluation and that the instrument content was appropriately targeted.

It is important to note that this study represents the first step in the validation process of the proposed scale, focusing on expert-based content analysis. Although this approach is recognized as a robust method for obtaining content-based validity evidence, it should be noted that consultation with the target population through focus groups was not conducted at this stage, which constitutes a methodological limitation. Such an approach could have contributed to a deeper understanding of item clarity, relevance, and acceptability from the perspective of end users.

Therefore, it should be emphasized that the instrument is not yet ready for clinical use or application in applied contexts. Subsequent stages of the validation process should include evaluation of internal structure, reliability, and convergent and discriminant validity, as well as applicability testing with the target population. Studies with representative samples and additional psychometric analyses will be essential to consolidate validity evidence and the practical usefulness of the acute vocal fatigue scale.

## CONCLUSION

The structure and items of the AFA-Voz Scale were considered excellent in terms of relevance/representativeness for the assessment of acute vocal fatigue. The final version of the instrument includes an anamnesis section to characterize the individual and their vocal demand, as well as numerical rating scales ranging from 0 to 10 points to judge three symptoms: fatigue, physical discomfort, and limitation in voice use.

## References

[1] Hunter EJ, Cantor-Cutiva LC, van Leer E, van Mersbergen M, Nanjundeswaran CD, Bottalico P (2020). Toward a consensus description of vocal effort, vocal load, vocal loading, and vocal fatigue. J Speech Lang Hear Res.

[2] Lei Z, Fasanella L, Martignetti L, Li-Jessen NY, Mongeau L (2020). Investigation of vocal fatigue using a dose-based vocal loading task. Appl Sci.

[3] Zambon F, Moreti F, Ribeiro VV, Nanjundeswaran C, Behlau M (2022). Vocal fatigue index: validation and cut-off values of the brazilian version. J Voice.

[4] Nanjundeswaran C, Shembel AC (2022). Laying the groundwork to study the heterogeneous nature of vocal fatigue. J Voice.

[5] Ijaz A, Yaqoob S, Mansoor AF, Aziz S, Masood F (2022). Association of vocal fatigue and years of experience in practicing speech and language pathologists: association of vocal fatigue and years of experience in pathologists. Pakistan Journal of Health Sciences.

[6] Stappenbeck LE, Bartel S, Brockmann-Bauser M (2022). Vocal fatigue as an indicator of complex voice disorders: a diagnostic and therapeutic challenge. HNO.

[7] Musial P, Dassie-Leite AP, Zaboroski AP, Casagrande RC (2010). Interferência dos sintomas vocais na atuação profissional de professores. Distúrb Comun.

[8] Torres RVNA, Lopes LW, Nascimento MA, Duarte JMT, Silva POC (2024). Phonatory tasks and outcome measures for assessing vocal fatigue: a scoping review. J Voice.

[9] Camargo MRMC, Zambon F, Moreti F, Behlau M (2019). Tradução e adaptação cultural e linguística da *Adapted Borg CR10 for Vocal Effort Ratings* para o português brasileiro. CoDAS.

[10] Pecorari A, Yamasaki R, Badaro F, Borrego MC, Behlau M (2022). Percepção de queixa vocal e autoavaliação do impacto de um problema de voz em atores profissionais de teatro. Audiol Commun Res.

[11] Pernambuco L, Espelt A, Magalhães HV, Lima KC (2017). Recomendações para elaboração, tradução, adaptação transcultural e processo de validação de testes em Fonoaudiologia. CoDAS.

[12] Lemos IO, Picanço Marchand DL, Oliveira Cunha E, Alves Silvério KC, Cassol M (2023). What are the symptoms that characterize the clinical condition of vocal fatigue? a scoping review and meta-analysis. J Voice.

[13] Polit DF, Beck CT (2006). The content validity index: are you sure you know what’s being reported? Critique and recommendations. Res Nurs Health.

[14] Alexandre NMC, Coluci MZO (2011). Validade de conteúdo nos processos de construção e adaptação de instrumentos de medidas. Cien Saude Colet.

[15] Zapata-Ospina JP, García-Valencia J (2022). Validity based on content: A challenge in health measurement scales. J Health Psychol.

[16] Hawker GA, Mian S, Kendzerska T, French M (2011). Measures of adult pain: Visual Analog Scale for Pain (VAS Pain), Numeric Rating Scale for Pain (NRS Pain), McGill Pain Questionnaire (MPQ), Short-Form McGill Pain Questionnaire (SF-MPQ), Chronic Pain Grade Scale (CPGS), Short Form-36 Bodily Pain Scale (SF-36 BPS), and Measure of Intermittent and Constant Osteoarthritis Pain (ICOAP). Arthritis Care Res.

