# Supplementary material for: Content-based validity evidence of the Acute Voice Fatigue Self-Perception Scale (AFA-Voz Scale)
Source: Codas. 2026 Apr 10;38(2):e20250060. doi: 10.1590/2317-1782/e20250060en (PMC13075840; doi:10.1590/2317-1782/e20250060en)
Supplement: Translation

# Translation: Evidências de validade baseada no conteúdo da Escala de Autopercepção da Fadiga Aguda para a Voz (Escala AFA-Voz)

**DOI:** 10.1590/2317-1782/e20250060pt

**Authors:** Priscila Oliveira, Mara Behlau

**Affiliations:** 1 Departamento de Fonoaudiologia, Universidade Federal da Paraíba – UFPB - João Pessoa (PB), Brasil.; 2 Universidade Federal de São Paulo – UNIFESP - São Paulo (SP), Brasil.; 3 Centro de Estudos da Voz – CEV - São Paulo (SP), Brasil.

**Keywords:** Voz, Fadiga, Autoavaliação, Sinais e Sintomas, Estudo de Validação

## Abstract

**Objetivo:**

Obter evidências de validade baseada no conteúdo da Escala de Autopercepção da Fadiga Aguda para a Voz (Escala AFA-Voz).

**Método:**

Esse estudo seguiu as diretrizes do Standards for Educational and Psychological Testing (SEPT) para validação de testes e foi realizado em duas etapas. Primeiro, o conteúdo foi elaborado a partir da análise de duas revisões sistemáticas, com elevada qualidade da evidência, para identificar os principais aspectos a serem avaliados na fadiga vocal aguda. Em seguida, foi constituído um comitê de cinco especialistas em voz – todos com mais de dez anos de experiência clínica e científica – para a avaliação da relevância dos itens selecionados, realizada por meio de uma escala do tipo likert em formulário online. Índices de validade de conteúdo geral (IVC) e por item (IVC-I) foram calculados para aferir a pertinência dos itens.

**Resultados:**

Os dados indicaram que o quadro clínico de fadiga vocal aguda pode ser avaliado em três dimensões: nível de fadiga autorrelatada, desconforto físico e restrição no uso da voz. Esses itens foram inseridos na escala, que também incluiu uma seção de anamnese para caracterização do paciente e sua demanda vocal. Na primeira rodada de avaliação, obteve-se um IVC de 0,96, com IVC-I variando entre 0,8 e 1,0. Após ajustes sugeridos, a segunda rodada apresentou IVC e IVC-I iguais a 1,00.

**Conclusão:**

Os itens da Escala AFA-Voz foram considerados excelentes quanto à relevância para avaliação da fadiga vocal aguda. A escala possui elevado potencial para uso clínico e em futuras pesquisas sobre o tema.

## INTRODUÇÃO

A produção vocal é uma atividade de elevada interdependência e sinergia muscular, habitualmente não extenuante. Contudo, quando utilizada em sobrecarga, pode haver dificuldade para mantê-la por longo tempo ou com qualidade adequada, revertendo-se em fadiga. A fadiga vocal é um sintoma mensurável, altamente individual, mais frequentemente referida por profissionais da voz, com impacto variável na qualidade do desempenho de diversas tarefas vocais^(1-3)^. Além de impactar significativamente a comunicação, também afeta a qualidade de vida e a atuação profissional destes indivíduos^(4).^

Por se tratar de um constructo complexo, não há consenso sobre a definição da fadiga vocal na literatura. No entanto, a maioria dos estudiosos concordam em defini-la como um fenômeno debilitante que envolve demandas vocais e respostas individuais a essas demandas, envolvendo sintomas individuais e diversas manifestações clínicas. Assim, a fadiga vocal pode ser caracterizada por um aumento percebido no esforço vocal e uma diminuição na qualidade da voz após uso prolongado da voz^(5,6)^, ou ainda como um declínio quantificável no desempenho fisiológico da produção da voz ou em sua percepção^(1)^.

O termo "fadiga vocal" tem sido utilizado de forma ampla para abranger manifestações sintomáticas variadas, sejam elas de natureza aguda, pontual ou crônica. A "fadiga vocal aguda" refere-se especificamente ao estado fisiológico e perceptivo que emerge após uma demanda vocal pontual e intensa, caracterizado por um limiar momentâneo de declínio no desempenho vocal, usualmente reversível com repouso vocal ^(1)^. A ausência do monitoramento e intervenção em quadros agudos de fadiga contribuem para a manutenção ou piora do quadro inicial, favorecendo a cronicidade desse sintoma, caracterizado pela “fadiga vocal crônica”, gerando implicações clinicas mais complexas e desencadeando maiores prejuízos comunicativos, econômicos e emocionais^(4,7)^.

A literatura científica dispõe de instrumentos amplamente reconhecidos para a autoavaliação de sintomas relacionados à fadiga vocal, tais como: a Escala de Esforço Fonatório Percebido (EFP), a Escala Borg CR-10, o Índice de Desvantagem Vocal (IDV), a Escala de Desconforto do Trato Vocal (EDTV) e o Índice de Fadiga Vocal (IFV)^(8)^. No entanto, esses instrumentos são limitados para oferecer uma mensuração específica da fadiga vocal aguda, ou seja, como resposta imediata a uma demanda, destacando a necessidade de métodos mais precisos para sua avaliação.

O EFP e a Escala Borg CR-10 são escalas subjetivas de esforço, utilizadas para quantificar a percepção do indivíduo sobre a demanda vocal^(9)^. No entanto, ambas não diferenciam com precisão o esforço vocal da fadiga vocal, uma vez que o esforço pode estar presente mesmo na ausência de fadiga, sendo influenciado por fatores como intensidade vocal e condição ambiental. O IDV é um questionário validado para avaliar a desvantagem percebida em decorrência de um problema de voz, mas não foi desenvolvido especificamente para mensurar fadiga vocal e não discrimina de forma detalhada a sensação de exaustão vocal decorrente do uso prolongado ou intenso da voz. A EDTV avalia desconfortos sensoriais na região laríngea, como ressecamento, ardência e tensão. No entanto, esses sintomas nem sempre refletem a presença de fadiga vocal, pois podem estar relacionados a outras condições, como refluxo laringofaríngeo ou tensão muscular extralaríngea^(10)^.

O IFV, por sua vez, foi especificamente idealizado para autoavaliação da fadiga vocal, mas pode não ser sensível para avaliar sua manifestação aguda. É capaz de capturar sintomas acumulados ao longo do tempo, com foco em aspectos perceptivos crônicos frequentemente associados ao uso vocal reiterado ao longo de dias ou semanas, mas não mudanças imediatas após uma demanda vocal exigente^(3)^. A fadiga vocal aguda representa uma resposta transitória, que se instala rapidamente após uma sobrecarga vocal específica e pode se manifestar por meio de alterações sutis na qualidade vocal, como aumento da rugosidade e maior esforço fonatório, achados que podem não ser captados com precisão pelo IFV.

Profissionais da voz podem sentir fadiga vocal aguda durante suas atividades cotidianas ou ao realizar tarefas vocais específicas, provocando limitações e incapacidade de curto prazo, especialmente em profissões que exigem o uso intensivo da voz, como professores, operadores de telemarketing, cantores e palestrantes. Mensurar e monitorar a percepção de fadiga aguda imediatamente após a performance pode fornecer informações valiosas sobre a resistência em manter o desempenho vocal, auxiliando no planejamento terapêutico adequado para manejo da fadiga, na manutenção do melhor rendimento possível do profissional da voz e na prevenção de distúrbios crônicos e/ou patologias laríngeas.

Não há instrumentos sensíveis e específicos para mensurar a fadiga vocal aguda percebida pelo próprio falante, na literatura atual. Dessa forma, o desenvolvimento de um instrumento direcionado à avaliação da fadiga vocal aguda representa um avanço para o rastreio precoce de alterações funcionais, o monitoramento da recuperação vocal e a proposição de intervenções terapêuticas mais precisas e imediatas.

O objetivo desse trabalho foi desenvolver e obter evidências de validade baseada no conteúdo da Escala de Autopercepção da Fadiga Aguda para a Voz (AFA-Voz), um instrumento para autoavaliação da fadiga vocal aguda.

## MÉTODO

Esse estudo foi direcionado à obtenção de evidência de validade baseada no conteúdo de um teste. Para isso, seguiu as diretrizes para validação de testes descritas por Pernambuco et al.^(11)^, que apresentaram uma série de recomendações baseadas nos princípios do *Standards for Educational and Psychological Testing* (SEPT), a partir de uma diretriz proposta por três organizações norte-americanas que compilou as mais sólidas recomendações e definições relacionadas aos aspectos psicométricos no processo de validação de instrumentos. Foi aprovado pelo comitê de ética para pesquisa com seres humanos (Parecer: 5.960.551).

Foram realizadas duas etapas: 1) elaboração do conteúdo; e 2) avaliação da relevância dos itens que compuseram o instrumento. Na etapa 1, foram consultadas duas revisões de escopo^(8,12)^, com elevado nível de qualidade da evidência, para auxiliar na seleção dos itens que poderiam compor o instrumento, a partir da definição do constructo. Foram selecionados itens que, de acordo com os estudos citados, podem auxiliar a caracterizar e mensurar a intensidade da fadiga vocal aguda.

Na etapa 2, foi selecionado um comitê de 5 especialistas em Voz, com mais de 10 anos de experiência clínica/científica, que foram informados sobre os objetivos da pesquisa e assinaram o termo de consentimento livre e esclarecido – TCLE e analisaram a versão inicial da escala proposta, em um formulário online. Os juízes foram solicitados a avaliar da relevância/representatividade dos itens por meio de uma escala do tipo *likert*, seguindo a seguinte legenda: 1 = o item não é relevante ou não é representativo; 2 = o item precisa de grande revisão para ser relevante ou representativo; 3 = o item precisa de uma pequena revisão para ser relevante ou representativo; 4 = o item é relevante ou é representativo. Caso marcasse as respostas 1, 2 ou 3 o juiz foi solicitado a incluir uma sugestão de revisão para que o item se tornasse relevante ou representativo.

Foram calculados o Índice de Validade de Conteúdo geral (IVC-T) e o Índice de Validade de Conteúdo por Item (IVC-I) para determinar o nível de concordância entre os juízes quanto à relevância ou representatividade dos itens do instrumento. O IVC é um método amplamente utilizado em estudos voltados para a avaliação da validade de conteúdo de um construto, pois mensura a proporção de juízes que concordam sobre o aspecto analisado no instrumento. Essa abordagem possibilita tanto a análise individual de cada item quanto a avaliação do instrumento em sua totalidade^(13,14)^.

Para calcular o IVC-I, foram considerados os escores atribuídos pelos avaliadores, a partir da seguinte fórmula:


$$\frac{IVC-I\;=número\;de\;respostas\;“3”\;ou\;“4”}{número\;total\;de\;respostas}$$ (1)

Já o IVC-T foi obtido a partir da média simples dos valores de IVC-I referentes à relevância dos itens. Ou seja, a soma de todos os IVC-I calculados separadamente, dividida pelo número de itens considerados na avaliação. Nesta pesquisa, adotou-se a seguinte classificação para a interpretação dos valores do IVC: excelente (IVC ≥ 0,90), bom (0,79 ≥ IVC ≤ 0,89) e ruim (IVC ≤ 0,78)^(14)^. Itens que apresentaram valores inferiores a 0,78 deveriam ser obrigatoriamente reformulados ou excluídos do protocolo.

## RESULTADOS

Na etapa de elaboração de conteúdo, a definição conceitual da fadiga vocal aguda foi estabelecida com base na literatura consultada. Definiu-se fadiga vocal aguda como um estado transitório, resultante de sobrecarga vocal recente, caracterizado por aumento do esforço fonatório e declínio da qualidade vocal, com recuperação esperada após repouso. Distingue-se da fadiga crônica por seu início súbito e curta duração^(1,4-6)^.

Já a definição operacional considerou as manifestações observáveis da fadiga vocal relatadas na literatura^(8,12)^ e a experiência dos autores. Foram selecionados três indicadores principais da fadiga vocal aguda: 1) nível de fadiga autorrelatado de forma imediata (b); 2) desconforto físico na região laríngea, como aperto, dor, entre outras (c); e 3) restrição no uso da voz (d), expressa por pausas frequentes ou redução na capacidade vocal. Esses três itens foram incluídos na configuração inicial do instrumento, empregando-se uma escala numérica (0-10 pontos) para julgamento da intensidade do sintoma^(8)^. Também foi adicionada uma seção de anamnese para breve caracterização do paciente e da sua demanda vocal (a).

Na etapa 2 de avaliação da relevância dos itens que compuseram o instrumento, foram realizadas 2 rodadas de julgamento com os especialistas. Na primeira rodada, o IVC foi igual a 0,96 e os valores de IVC-I foram a=0,8; b=1,0; c=1,0; d=1,0; indicando relevância excelente. Nas duas rodadas nenhum item foi considerado irrelevante, mas alguns ajustes foram sugeridos para melhor clareza e objetividade dos itens do instrumento. No item (a), referente à seção de anamnese, já que incluía data de coleta, nome do indivíduo, profissão, tempo de uso profissional da voz, tipo de demanda vocal (falada, cantada ou ambos), foi sugerida a inclusão de itens sobre a intensidade da última atividade vocal realizada (forte, habitual e fraca) e duração da última atividade vocal realizada (em minutos). Nos itens (c) e (d) houve uma breve reformulação na descrição do item e a inserção de possíveis exemplos para auxiliar a percepção dos sintomas por parte do paciente, melhorando a interpretação e aplicabilidade do instrumento.

Além de todas as sugestões dos juízes, foi incorporada uma instrução definida pelas próprias autoras, para orientar o respondente quanto ao preenchimento do questionário. Dessa forma, o instrumento final foi composto por uma breve seção de anamnese, contendo 5 perguntas sobre a profissão e características da demanda vocal do respondente, além de 3 itens que abordaram a avaliação da fadiga vocal aguda propriamente dita.

Após finalização da segunda rodada, e com a realização de todos os ajustes sugeridos, o instrumento foi reavaliado e todos os valores de IVC e IVC-I foram iguais a 1,00 (100,0%), considerada como uma concordância excelente. A versão final do instrumento foi apresentada no Quadro 1.

**Quadro 1:** Versão final da Escala de Autopercepção da Fadiga Aguda para a Voz (Escala AFA-Voz)

**ESCALA DE AUTOPERCEPÇÃO DA FADIGA AGUDA PARA A VOZ (Escala AFA-Voz)**									
**Oliveira e Behlau, 2026**									
Nome: _______________________________________________________ Data: ___/___/_____									
Profissão: ____________________________________________________ D.N.: ___/___/_____									
Há quanto tempo usa a voz profissionalmente: _________________________________________									
Tipo de demanda vocal: ( ) Falada ( ) Cantada ( ) Falada e Cantada									
Intensidade da última atividade vocal realizada: ( ) Forte ( ) Habitual ( ) Fraca									
Duração da última atividade vocal realizada (em minutos): ________________________________									
**Instrução: Avalie como você percebe sua voz nesse momento e marque o número que mais representa sua condição atual em relação aos sintomas abaixo:**									
1) **Fadiga**									
De 0 a 10, o quanto você sente sua voz cansada/fadigada agora?									
**1**	**2**	**3**	**4**	**5**	**6**	**7**	**8**	**9**	**10**
Nenhuma fadiga					Máxima fadiga				
*2)* **Desconforto físico**									
De 0 a 10, o quanto você sente de desconforto no pescoço/garganta para produzir a voz agora? *(Por exemplo: aperto, dor, ardência, entre outros.)*									
**1**	**2**	**3**	**4**	**5**	**6**	**7**	**8**	**9**	**10**
Nenhum desconforto					Máximo desconforto				
*3)* **Limitação para usar a voz**									
De 0 a 10, o quanto você sente que sua voz está limitada agora? *(Por exemplo: dificuldade para variar o tom da voz, dificuldade para falar forte, dificuldade para falar fraco, dificuldade para projetar a voz, dificuldade para manter o som, a qualidade ou o timbre da voz, dificuldade para manter o som da voz, dificuldade para continuar falando, dificuldade para continuar cantando, entre outros.)*									
**1**	**2**	**3**	**4**	**5**	**6**	**7**	**8**	**9**	**10**
Nenhuma limitação					Máxima limitação				

## DISCUSSÃO

Na área da saúde, o adequado processo de avaliação de um indivíduo depende da análise de sua percepção sobre algum aspecto específico, o que pode ser feito com utilização de testes, cujas interpretações dos resultados sejam válidas, confiáveis/precisas e equitativas. A validade de conteúdo é uma etapa que se refere à reunião de evidências sobre o tema, redação, formato, tarefas ou questões de um teste, bem como as instruções para os procedimentos necessários para administrá-lo e pontuá-lo^(11)^. Trata-se de uma etapa essencial no desenvolvimento de instrumentos de avaliação, pois garante a relevância e abrangência na mensuração dos construtos, resultando em uma medida quantificável de validade^(11,15)^.

Nessa pesquisa, a definição conceitual e operacional da fadiga vocal aguda representou uma etapa essencial no desenvolvimento do instrumento, sendo fundamentada na literatura^(8,12)^ e na experiência clínica das autoras. A delimitação do construto, diferenciando-o da fadiga vocal crônica, permitiu estabelecer parâmetros mais sensíveis para a detecção de sinais agudos, de instalação rápida e reversíveis com o repouso vocal. A vasta investigação realizada nos artigos de referência, permite afirmar que os resultados encontrados refletem uma síntese dos achados das pesquisas mais proeminentes e confiáveis sobre o tema.

Já a escolha da escala numérica de 0 a 10 pontos se deu em virtude da sua praticidade, sensibilidade e ampla aceitação desse formato para mensurar sintomas subjetivos, como a fadiga vocal. Em comparação à escala do tipo Likert, voltada à mensuração de atitudes ou percepções, a escala numérica permite captar variações graduais na intensidade do sintoma. E, embora a Escala Visual Analógica (EVA) também seja sensível para esse objetivo e referida por grande parte dos estudos, sua aplicação pode ser limitada devido à necessidade de suporte visual e interpretação mais abstrata, o que é capaz de comprometer a precisão da resposta. A escala numérica, por sua vez, é de fácil compreensão e aplicação até mesmo em ambientes não clínicos, mantendo boa precisão na avaliação^(16)^.

As sugestões dos especialistas oferecidas nas rodadas de julgamento foram incorporadas de forma a aprimorar a clareza e aplicabilidade do instrumento. A inserção de novos itens na anamnese, por exemplo, permitiu caracterizar melhor o contexto de uso vocal recente, enquanto os ajustes na redação e a inclusão de exemplos, especialmente no item 3, favoreceram a compreensão dos itens por parte dos juízes. Além disso, a adição de uma instrução preliminar contribuiu para padronizar o preenchimento e reduzir possíveis ambiguidades na interpretação dos itens.

Apesar do instrumento ter obtido uma avaliação satisfatória na versão inicial, a realização de tais ajustes sugeridos refletiram a alta concordância entre os juízes quanto à relevância dos itens (IVC = 1,00) na segunda rodada, sinalizando que os indicadores selecionados (autorrelato imediato de fadiga, desconforto físico na laringe e restrição no uso da voz) foram considerados representativos do construto avaliado e que o conteúdo foi bem direcionado.

É importante mencionar que esse estudo representa o primeiro passo do processo de validação da escala proposta, centrada na análise de conteúdo por especialistas. Ainda que essa abordagem seja reconhecida como um método robusto para obtenção de evidências de validade baseada no conteúdo, cabe destacar que a consulta à população-alvo por meio de grupos focais não foi realizada nesta fase, o que configura uma limitação metodológica. Tal abordagem poderia ter contribuído para uma compreensão mais profunda da clareza, pertinência e aceitabilidade dos itens sob a perspectiva dos usuários finais.

Dessa forma, ressalta-se que o instrumento ainda não está pronto para uso clínico ou em contextos aplicados. Etapas subsequentes do processo de validação deverão contemplar a avaliação da estrutura interna, da confiabilidade e da validade convergente e discriminante, bem como testes de aplicabilidade com a população-alvo. Estudos com amostras representativas e análises psicométricas adicionais serão fundamentais para consolidar as evidências de validade e utilidade prática da escala de fadiga vocal aguda.

## CONCLUSÃO

A estrutura e os itens da Escala AFA-Voz foram considerados excelentes quanto à relevância/representatividade para avaliação da fadiga vocal aguda. A versão final do instrumento apresenta uma seção de anamnese para caracterização do indivíduo e da sua demanda vocal, e escalas numéricas de 0 a 10 pontos para julgamento de três sintomas: fadiga, desconforto físico e limitação para usar a voz.
